# Deletion of p75^NTR^ rescues the synaptic but not the inflammatory status in the brain of a mouse model for Alzheimer’s disease

**DOI:** 10.3389/fnmol.2023.1163087

**Published:** 2023-05-05

**Authors:** Hendrik Demuth, Shirin Hosseini, Henning Peter Düsedeau, Ildiko Rita Dunay, Martin Korte, Marta Zagrebelsky

**Affiliations:** ^1^Division of Cellular Neurobiology, Zoological Institute, Braunschweig, Germany; ^2^Research Group Neuroinflammation and Neurodegeneration, Helmholtz Centre for Infection Research, AG NIND, Braunschweig, Germany; ^3^Institute of Inflammation and Neurodegeneration, Health Campus Immunology, Infectiology and Inflammation (GC-I3), Otto-von- Guericke University, Magdeburg, Germany; ^4^Center for Behavioral Brain Sciences (CBBS), Magdeburg, Germany

**Keywords:** Alzheimer’s’s disease, neuroinflammation, p75 neurotrophin receptor, synaptic plasticity, spatial learning, cognitive decline, flow synaptometry, GluR1

## Abstract

**Introduction:**

Alzheimer’s disease (AD), is characterized by a gradual cognitive decline associated with the accumulation of Amyloid beta (Aβ)-oligomers, progressive neuronal degeneration and chronic neuroinflammation. Among the receptors shown to bind and possibly transduce the toxic effects of Aβ-oligomers is the p75 neurotrophin receptor (p75^NTR^). Interestingly, p75^NTR^ mediates several crucial processes in the nervous system, including neuronal survival and apoptosis, maintenance of the neuronal architecture, and plasticity. Furthermore, p75^NTR^ is also expressed in microglia, the resident immune cells of the brain, where it is markedly increased under pathological conditions. These observations indicate p75^NTR^ as a potential candidate for mediating Aβ-induced toxic effects at the interface between the nervous and the immune system, thereby potentially participating in the crosstalk between these two systems.

**Methods:**

Here we used APP/PS1 transgenic mice (APP/PS1tg) and compared the Aβ-induced alterations in neuronal function, chronic inflammation as well as their cognitive consequences between 10 months old APP/PS1tg and APP/PS1tg x p75^NTRexonIV^ knockout mice.

**Results:**

Electrophysiological recordings show that a loss of p75^NTR^ rescues the impairment in long-term potentiation at the Schaffer collaterals in the hippocampus of APP/PS1tg mice. Interestingly, however loss of p75^NTR^ does not influence the severity of neuroinflammation, microglia activation or the decline in spatial learning and memory processes observed in APP/PS1tg mice.

**Conclusion:**

Together these results indicate that while a deletion of p75^NTR^ rescues the synaptic defect and the impairment in synaptic plasticity, it does not affect the progression of the neuroinflammation and the cognitive decline in a mouse model for AD.

## Introduction

Alzheimer’s disease (AD) is one of the most common progressive neurodegenerative disorders and a leading cause of dementia in the human population. According to the amyloid-β (Aβ) hypothesis, the initiating factors triggering the self-sustaining inflammation are small Aβ-oligomers (Aβo) binding to a plethora of cellular receptors on various cell types of the nervous system, thereby inducing toxic effects at the cellular level (Salminen et al., 2008; Mroczko et al., 2018). The p75 neurotrophin receptor (p75^NTR^) binds Aβo directly (Fombonne et al., 2009). Interestingly, this member of the tumor necrosis factor receptor superfamily is expressed on various cell types of the central nervous system (CNS) (Yamamoto et al., 1998; Kim et al., 2006) including neurons (Mufson et al., 1992), microglia (Düsedau et al., 2019), and astrocytes (Chen et al., 2020). The p75^NTR^ has been found to serve multiple, in parts opposing, functions (Meeker and Williams, 2015). For instance, whereas the association of mature neurotrophins with the receptor can facilitate neuronal survival and growth (Hempstead et al., 1991), binding of the respective proforms induces apoptotic pathways (Friedman, 2000; Beattie et al., 2002). The neuronal p75^NTR^ expression is massively increased in the brain of AD patients (Mufson and Kordower, 1992). The functional relevance of p75^NTR^ expression in the AD context was shown in several *in vitro* studies where treatment of hippocampal neurons with Aβo resulted in p75^NTR^-mediated cell death (Sotthibundhu et al., 2008). Furthermore, p75^NTR^ has been shown to regulate neuronal architecture and activity-dependent synaptic plasticity in the mature hippocampus (Rösch et al., 2005; Woo et al., 2005; Michaelsen et al., 2010; Zhang et al., 2012). Indeed, p75^NTR^ negatively regulates dendritic complexity and spine density in healthy hippocampal neurons (Zagrebelsky, 2005). Deletion of p75^NTR^ rescues the Aβo-induced dendritic spine loss in primary hippocampal neurons and the severity of spine loss is correlated with the amount of p75^NTR^ localization at synapses (Patnaik et al., 2020). Importantly, p75^NTR^ is involved in regulating synaptic plasticity, which is the cellular correlate of information storage in the brain (Korte and Schmitz, 2016). Genetic ablation of p75^NTR^ leads to impaired long-term depression (Rösch et al., 2005; Woo et al., 2005). In addition to its regulatory effects on the structure and function of the neuronal network, p75^NTR^ is also implicated in a variety of immune functions making it an interesting molecule in the context of AD-related chronic neuroinflammation. Indeed, p75^NTR^ directly regulates the functions of plasmacytoid dendritic cells, thereby increasing allergen-specific T-cell proliferation and cytokine production in a mouse model of asthma (Bandoła et al., 2017). Various CNS region-specific studies revealed increased expression of p75^NTR^ on CNS-resident immune cells during different insults. Infection-related insults result in a strong microglial p75^NTR^ upregulation, e.g., upon *Toxoplasma gondii* infection-induced neuroinflammation (Düsedau et al., 2019) or *Streptococcus pneumoniae*-induced meningitis (D. Zhang et al., 2021). Importantly, also chronic inflammation in the 5xFAD AD mouse model is associated with increased p75^NTR^ expression in microglia (Capsoni et al., 2017). Whether a p75^NTR^ knockout is ameliorating AD pathology in mouse models is still critically discussed. Results reporting beneficial effects were obtained by blocking p75^NTR^ in Tg2576 mice (Simmons et al., 2014) and upon conditional deletion of p75^NTR^ specifically from basal cholinergic forebrain neurons (Qian et al., 2018). On the other side, ubiquitous deletion of p75^NTR^ did not significantly affect the cognitive performance of APP/PS1 transgenic mice (APP/PS1tg) (Wang et al., 2011), leaving open the specific role of p75^NTR^ in different aspects of AD pathogenesis.

Besides the typical pathological hallmarks including Aβ plaque deposition, neurofibrillary tangles, synapse loss, and gliosis severely affected brain areas display progressively increasing neuroinflammation (Rao et al., 2012; Deture and Dickson, 2019; for a recent review see Scheiblich et al., 2020). Indeed, various approaches to block neuroinflammation in mouse models of AD revealed that chronic inflammation is the main driver of AD progression (Heneka et al., 2013; Lonnemann et al., 2020). Emerging evidence suggests that chronically activated microglia together with brain-resident and infiltrating peripheral immune cells perturb the homeostatic state of the brain parenchyma and contribute to synaptic loss, ultimately affecting neuronal plasticity and resulting in progressive cognitive impairment (Hatanpää et al., 1999; Rao et al., 2012; Heneka et al., 2013, 2015). Thus, deciphering the molecules and mechanisms mediating the effects of inflammation on the neuronal network is crucial for understanding the pathogenesis of AD.

Taken together, the expression of p75^NTR^ on cells of the nervous and immune system in the brain combined with its pleiotropic actions in regulating neuronal plasticity and immune response identify it as a potential molecule mediating the interplay between neurons and inflammation in the pathogenesis of AD. Thus, in this study, the effects of a p75^NTR^ deletion in APP/PS1tg mice were assessed simultaneously on hippocampal activity-dependent synaptic plasticity, synaptic structure, immune activation, and cognitive performance.

## Materials and methods

### Animals

10 months old female and male C57BL/6 J WT, APPswe x PS1ΔE9 (Jankowsky et al., 2004) (APP/PS1tg), p75^NTRexonIV^KO (von Schack et al., 2001) (p75^NTR^ KO), APP/PS1tg x p75^NTR^ KO mice were used in this study. Mice were bred and kept under standard housing conditions at the animal facility of the Technical University of Braunschweig, Germany. All experimental procedures were approved by the animal welfare representative of the TU Braunschweig and the LAVES (Oldenburg, Germany, Az. §4 (02.05) TSchB TU BS und Az.33.19-42,502-04-20/3498).

### Electrophysiological recordings

Brains from 10 months old mice were rapidly isolated and kept in ice-cold carbonated (95% O_2_ and 5% CO_2_) artificial cerebrospinal fluid (ACSF) containing (in mM) 124 NaCl, 4.9 KCl, 1.2 KH_2_PO_4_, 2.0 MgSO_4_, 2.0 CaCl_2_, 24.6 NaHCO_3_, and 10 D-glucose, pH 7.4. The hippocampi were dissected and 400 μm thick hippocampal slices were cut using a manual tissue slicer (Stoelting). The acute hippocampal slices were rapidly placed in an interface recording chamber (Scientific System Design), where they were kept to equilibrate at 32°C with a constant flow rate (0.5 ml/min) of carbonated ACSF for 2 h before recording.

Field excitatory postsynaptic potentials (fEPSPs) were measured in the stratum radiatum of the CA1 hippocampal sub region upon electrical stimulation of the CA3 to CA1 Schaffer collateral pathway using a monopolar, lacquer-coated stainless steel electrode (5 MΩ; AM Systems). For recording fEPSPs (defined as the first slope function), the recording electrode (5 MΩ; AM Systems) was placed in the apical dendritic layer of CA1 at least 20 μm away from the stratum pyramidale. The recorded signals were amplified with a differential amplifier (model 1700, AM Systems) and digitized using a CED 1401 analog-to-digital converter (Cambridge Electronic Design). After pre-incubation, a synaptic input–output curve (afferent stimulus intensity versus fEPSP slope) was generated to measure basal synaptic transmission. Test stimulation intensity was adjusted to elicit an fEPSP slope of 40% of the maximal fEPSP response. To examine short-term plasticity, a paired-pulse stimulation protocol was performed in which two consecutive stimuli of equal intensity were delivered at different interpulse intervals of 10, 20, 40, 60, 80, and 100 ms. To investigate long-term potentiation (LTP), LTP was elicited 20 min after recording a stable baseline by theta-burst stimulation (TBS), including four bursts at 100 Hz repeated 10 times at an interval of 200 ms. This stimulation was repeated three times at 10 s intervals. The slope of the fEPSPs was measured over 60 min and normalized to baseline. Data acquisition and offline analysis were performed using IntraCell software (version 1.5, LIN, Magdeburg, 2000) (Hosseini et al., 2021).

### FACS analysis

Fluorescence-Activated Cell Sorting (FACS) was performed to analyze the activation status of microglial cells as described elsewhere (Lonnemann et al., 2020). Briefly, at 10 months of age, mice were deeply anesthetized with CO_2_ and sacrificed *via* decapitation. After dissection of the brain, a single cell suspension was created using the Adult Brain Dissociation Kit (Miltenyi Biotec Order no. 130-107-677) and the GentleMACS (Milteny). Cells were resuspended in FACS staining buffer (PBS + 1% FCS + 0.1% Na-Azide) and stained for 30 min with the following antibodies: anti-CD11b-PerCP-cy5.5 (1:50), anti-CD45-APC (1:50), and anti-CD68-PE (1:50) in a 96-well plate. The cells were measured using the BD LRS II SORP and analyzed with FlowJo Software (version 10.8.0). First, cells were gated for CD11_intermediate_ and CD45 _intermediate_ expression. Based on the resulting cell population, CD68-positive cells were defined as cells above a fluorescence threshold of 10^3^ for the CD68 marker.

### ELISA

To quantify the expression of the cytokines IL-1β, CCL2, and IL-10, ELISA was performed. The mice were deeply anesthetized with CO_2_ and killed *via* decapitation. One hemisphere was frozen in liquid nitrogen. On the day of the experiment, the tissue was homogenized in 400 μl STKM lysis buffer (250 mM sucrose, 50 mM Tris–HCl, 25 mM KCl, and 5 mM MgCl_2_, protease inhibitor mixture; cOmplete®) using the GentleMACS tissue homogenizer according to protocol. The samples were centrifuged at 4°C for 10 min at 13,000 × *g*, and the supernatant was collected. To determine cytokine levels, mouse IL-1β, CCL2, and IL-10 ELISA kits (R&D Systems, IL-1β: DY401; IL-10: DY417, CCL2: DY479) were used according to the manufacturer’s recommendations. Absorbance at 450 nm was measured with an Epoch microplate reader from BioTek. Analysis was conducted using the Gen5 software.

### Morphological analysis of hippocampal neurons: Golgi Cox staining

For the analysis of the dendritic spine density, hippocampal neurons were stained using the Golgi-Cox method. Mice were deeply anesthetized with CO_2_ and sacrificed *via* decapitation. After dissection, the left hemisphere was incubated in an FD rapid Golgi-Cox staining kit according to the manufacturer’s guidelines. Afterward, the hemispheres were embedded in 2% agar and cut into 200 μm thick coronal sections using a vibratome (Leica, VT 1000 S). The slices were mounted on gelatine-coated glass slides and allowed to dry. The final development was conducted according to the manufacturer’s protocol. Slices were mounted using Permount (Thermo Fisher Scientific).

### Immunofluorescent labeling

Immunofluorescence (IF) was performed to assess the region-specific microglial activation. Briefly, mice were deeply anesthetized with CO_2_ and sacrificed *via* decapitation. The brain was post-fixed for 24 h with 4% PFA in PBS at 4°C followed by a cryoprotection step for 72 h in 30% sucrose in PBS. Afterward, the brains were frozen in Tissue-TEK® compound (A. Hartenstein Laborversand) and stored at -70°C. For IF, the brains were cut into 30 μm thick slices using a Leica VT 100 Cryostat. In total, five successive sections from each hemisphere were collected in each well and were washed three times with a solution containing 0.1% Triton-X100 (Applichem) in PBS for 5 min. Next, the sections were incubated for 1 h at room temperature in a blocking and permeabilizing solution containing 0.3% of TritonX-100, 5% of goat serum, 5% of donkey serum, and 5% bovine serum albumin (BSA) in PBS. In the next step, sections were incubated overnight at 4°C with rabbit anti-ionized calcium-binding adaptor molecule 1 (Iba1; WAKO 1:1000, AB_839504), rat anti-CD68 clone FA-11 (Bio-Rad,1:800, AB_322219), and mouse anti-Amyloid-β (Sigma Aldrich, 1:1000, BAM-10 clone, A5213) antibodies diluted in blocking solution. After 30 min washing with PBS, the sections were incubated with Cy™2-conjugated AffiniPure Goat Anti-rabbit IgG (H + L) (Jackson Immuno research, 1:500, AB_2338006), Cy™3-conjugated AffiniPure Donkey Anti-mouse IgG (H + L) (Jackson Immuno research, 1:500, AB_2340823), and Cy™5-conjugated AffiniPure Goat Anti-Rat IgG (H + L) (Jackson Immuno research, 1:500, AB_2338264) antibodies diluted in 0.05% TritonX-100 in PBS for 2 h at room temperature. Afterward, the sections were washed thoroughly with PBS for 30 min, stained with 4′,6-diamidino-2-phenylindole (DAPI) (Sigma-Aldrich), and mounted with Fluoro-gel medium (Electron Microscopy Sciences, Hatfield, PA).

### Imaging and analysis

For the region-specific analysis of microglia activation, the CA1 area of brain slices stained for Iba1 and CD68 and z-stacks were acquired using a ZEISS Imaging system equipped with an ApoTome module and a 20x objective [1.25 Numerical aperture (NA)] at 0.5 μm increments. Intensity and exposure time were set using slices of three WT mice, averaged, and kept constant throughout imaging for all groups. Analysis was conducted using the IMARIS® software (Bitplane). Briefly, the surface of single microglial cells was reconstructed from the original image (surface grain size = 0.45 μm, the diameter of largest sphere = 1.70 μm, number of voxels between 1,57e4 – 6,59e4). Nuclei models generated from the DAPI staining (surface grain size = 0.45 μm, the diameter of largest sphere = 1.70 μm, and the number of voxels above 493) were used to exclude cells that could not be differentiated as single microglial cells. The original staining was masked and used to construct filament trees based on the single-cell surfaces (Dendrite starting point diameter = 11,4 μm, Dendrite seed point diameter = 0.681 μm). The intensity of the CD68 labeling, dendritic complexity (by the Sholl analysis), number of branch points, and number and volume of cells were taken from the data automatically provided by the IMARIS software.

### Synaptosome isolation

Synaptosomes were isolated as described elsewhere (Düsedau et al., 2021). In brief, frozen hippocampus brain samples were homogenized according to standard protocols (Gray and Whittaker, 1962; Kolodziej et al., 2016) but with slight modifications. After sucrose density centrifugation, the crude synaptosome pellet (P2) was resuspended in SET buffer (320 mM sucrose, 1 mM EDTA, 5 mM Tris, pH 7.4) with 5% DMSO, aliquoted, and slowly frozen to -80°C using an isopropanol freezing container, and stored until further use (Hobson and Sims, 2019).

### Synaptosome staining and flow synaptometry

The synaptosomes were stained and analyzed according to a method previously described (Düsedau et al., 2021). Aliquots of frozen synaptosomes were thawed in a water bath at 37°C and centrifuged for 10 min at 25,000 x *g* to remove the sucrose-containing buffer. The supernatant was aspired gently and the pellets were resuspended in FoxP3 Transcription Factor Staining Buffer (eBioscience) and incubated on ice for 45 min. Subsequently, the samples were centrifuged again for 10 min at 25,000 x g, resuspended in Permeabilization Buffer (eBioscience), reconstituted with 10 % Normal Goat Serum (NGS, ThermoFisher), and stained with primary antibodies against Gephyrin (#ab136343, Abcam, UK), GluR1 (ABN241; Sigma-Aldrich), Homer1 (#MAB6889, R&D Systems, MN, United States), Synaptophysin1 (#101004, Synaptic Systems), and VGLUT1 (#135303, Synaptic Systems). Following incubation, the samples were washed and resuspended in Permeabilization buffer +10% NGS and stained with matching secondary antibodies: goat anti-mouse AlexaFluor® 405 (#A31553, ThermoFisher), goat anti-rabbit AlexaFluor® 488 (#ab150081, Abcam), goat anti-chicken AlexaFluor® 546 (#A-11040, Abcam), and goat anti-guinea pig AlexaFluor® 647 (#A21450, Thermo Fisher). Finally, the samples were washed again and resuspended in SET buffer before adding the styryl dye FM4-64 (#T13320, ThermoFisher) to a final concentration of 0.2 μg/mL (Hobson and Sims, 2019). The samples were measured using the Attune NxT Flow Cytometer (ThermoFisher) equipped with 405, 488, 561, and 633 nm lasers. Voltages for forward-scatter light (FSC), side-scatter light (SSC), and fluorescence detection channels were set as follows: FSC 400 V, SSC 500 V, VL1 400 V, BL1 400 V, BL3 380 V, YL1 400 V, and RL1 440 V. For optimal acquisition of synaptosomes, the FSC-triggered detection was replaced by a fluorescence-triggered detection with FM4-64 in the BL3 channel (threshold set to 0.3 × 10^3^ to select only FM4-64-positive events). Furthermore, the event rate was kept below 300 events/s by utilizing the slowest flow rate in combination with an adequate dilution of the sample prior to measurement to reduce coincident particle detection. A size range from 300-1,000 nm was applied to detect events in the FSC channel using red-fluorescent silica beads with a diameter of 300 nm (#DNG-L020, Creative Diagnostics, NY, United States) and 1,000 nm (#DNG-L026, Creative Diagnostics) (Hobson and Sims, 2019). Obtained data were analyzed using FlowJo software (Version 10, FlowJo LLC, OR, USA). Fluorescence Minus One (FMO) controls were used to determine the level of autofluorescence.

### Behavioral experiments

All behavioral tests were conducted during the light cycle under dim light illumination. The test repetitions were performed at the same time of day. All experiments were performed by an experimenter blind to the mouse genotypes.

### Open field test

The open-field test was performed to test for anxiety-like and explorative behavior as previously described (Walsh and Cummins, 1976; Hosseini et al., 2018). Briefly, the mice were placed into the open field apparatus (40x40x40cm) along one side and allowed to explore the area for 5 min. To avoid any odor cues, the box was cleaned with 70% ethanol after each mouse. During the test, the mouse trajectories were acquired using a digital camera positioned above the maze and the tracking software ANY-maze (Stoelting). The software recorded the total distance traveled, average speed, and percentage of activity in the periphery (subdivided in corner and border zone) and center. The center part of the arena was defined by a 10 × 10 cm square.

### Elevated plus maze test

Assessment of anxiety behavior was conducted using the Elevated Plus maze as described previously (Pellow et al., 1985). In this test, the apparatus consists of a cross with two opposed open arms (25 × 5 cm) and two opposed closed arms surrounded by 20 cm high walls (25 × 5 cm). The arena was elevated 50 cm above the floor. Mice were placed in the central part of the arena (5 × 5 cm) facing toward an open arm and permitted to explore the maze freely for 5 min. During the test, the mouse trajectories were acquired using a digital camera positioned above the maze and the tracking software ANY-maze (Stoelting).

### Barnes maze task

The Barnes Maze test was used to assess spatial learning and memory formation (Barnes, 1979). In this test, a 90 cm circular platform containing 20 equally spaced holes around its perimeter was positioned 120 cm above the ground. A dark escape box was placed under one of the holes and three visual cues were attached to the walls surrounding the maze guiding the mice in their spatial navigation toward the escape box. The mice were tracked with an overhead digital camera and the ANY-maze software (Stoelting). Before starting the actual training in the Barnes Maze, a habituation period was conducted to reduce stress during the test. Furthermore, the mice were observed to ensure that motility and vision were intact and similar in all groups. During this phase, the mice were free to explore the platform for 30 s. At the end of this period, if they did not find the escape hole, the mice were guided to the escape box and allowed to remain in the box for 2 min before being returned to their home cage. Subsequently, 4 days of Barnes maze training were performed. Each training day consisted of 4 trials in which the mice were placed in a circular black box in the middle of the maze to randomize their starting direction. After 15 s, the box was lifted, and mice were permitted to freely explore the maze for a total amount of 180 s or until they entered the escape box. Distal cues in the room were obscured by a white curtain that encircled the platform. If the task was not solved, the mice were guided to the escape box and allowed to remain inside for 60 s. To evaluate the short- and long-term reference memory, two probe trial tests were performed on day 5 (short-term) and day 12 (long-term). During the probe trials, the escape box was removed and the mice were allowed to explore the maze for 45 s. To avoid odor stimuli, the platform was cleaned with 70% ethanol after each mouse and was randomly rotated between all trials and tests.

### Statistical analysis

Data were analyzed and plotted using the GraphPad Prism 8 software and are presented as mean ± SEM. Results of electrophysiology were subjected to ordinary one-way (for the potentiation between 55 and 60 min after induction) and two-way repeated measures ANOVA in combination with Bonferroni posttest (for the evaluation of the input/output curves, the paired-pulse facilitation, and the LTP curves). Results of the spine density analysis were subjected to one-way ANOVA with Fisher’s LSD *post hoc* test. Data on cytokine secretion were subjected to a one-way ANOVA with Tukey’s *post hoc* test. Two-way repeated measures ANOVA (for the evaluation of the learning in the Barnes maze) and one-way repeated measures ANOVA (for all other behavioral tests) were used in the behavioral experiments. The posttest performed and the number of animals (*n*); brain slices, cells, and samples are given in the results section and figure legends, respectively. In all graphs the data are shown as mean ± standard error of the mean (SEM), and the statistical significance was indicated as follows: ^*^ = *p* < 0.05, ^**^ = *p* < 0.01, and ^***^ = *p* < 0.001. All experiments were evaluated by an experimenter blind to the genotype of the mice.

The minimum number of mice to be used in each experiment was calculated *a priori* using G∗Power 3.1.9.4 software (Heinrich Heine University Düsseldorf, Germany).

## Results

### Deletion of p75^NTR^ rescues the impaired long-term potentiation in APP/PS1 transgenic mice

**Figure 1:**
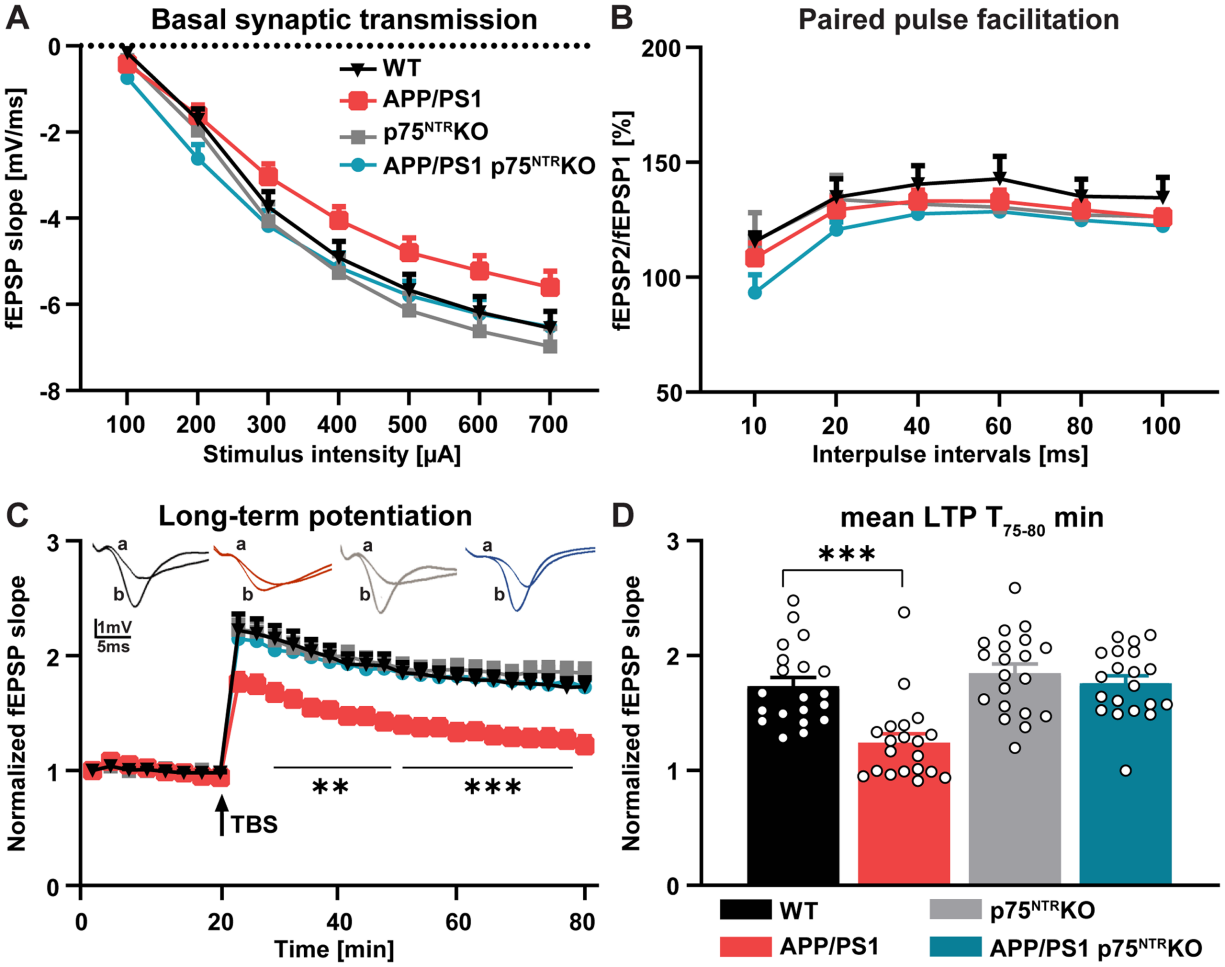
Deletion of p75^NTR^ rescues the impaired long-term potentiation (LTP) in APP/PS1tg mice**. (A)** Input–output curves of field excitatory postsynaptic potential (fEPSP) slopes in hippocampal slices obtained from all tested groups did not show any significant differences [*N* (number of animals in each group) = 4, *n* (number of slices in each group) = 20]. **(B)** The paired-pulse facilitation (PPF) of the fEPSP slopes given as the response to the second stimulation over the first one at different interpulse intervals (10, 20, 40, 60, 80, and 100 ms) in hippocampal slices did not exhibit any significant differences between the groups (*N* = 4, *n* = 16). **(C)** The hippocampal slices obtained from APP/PS1tg mice showed significantly lower LTP compared to controls. Remarkably, the impairment of LTP was completely abolished in APP/PS1tg x p75^NTR^ knockout mice. **(D)** APP/PS1tg mice exhibited significantly reduced maintenance (T 75–80 min) of LTP compared to controls, whereas p75^NTR^ deletion completely rescued this deficit to the control level (*N* = 4, *n* = 20). Data are presented as mean ± SEM, two-way RM ANOVA **(A-C)**, and ordinary one-way ANOVA **(D)** of data, and *post hoc* Tukey’s multiple-comparisons test were performed. ^***^p < 0.001 compared to the control.

To address the role of the p75^NTR^ in the pathology of Alzheimer’s disease (AD), we started by assessing whether a complete deletion of p75^NTR^ rescues the well-described deficits observed in activity-dependent synaptic plasticity (Chapman et al., 1996; Trinchese et al., 2004) in the hippocampus of APP/PS1 transgenic (APP/PS1tg) mice (Gengler et al., 2010), a commonly used mouse model for AD. Therefore, different parameters of basal synaptic transmission and activity-dependent synaptic plasticity were compared at the CA3-CA1 Schaffer collateral pathway in 10 months old WT, APP/PS1tg, p75^NTR^ KO, and APP/PS1tg x p75^NTR^ KO mice. First, to assess changes in basal synaptic transmission, the size of the field excitatory postsynaptic potential (fEPSP) slope at increasing stimulus intensities was compared as input/output curves. With this approach, no significant difference could be observed between the experimental groups (Two-way RM ANOVA: *F* (3, 76) = 2.407, *p* = 0.074); however, APP/PS1tg mice showed a trend toward a decrease in the input/output curve which was not present in APP/PS1tg x p75^NTR^ KO and control mice (Figure 1A). Next, short-term plasticity at the CA3-CA1 Schaffer collateral pathway was investigated using paired-pulsed facilitation (PPF) and revealed no significant difference between the groups (Two-way RM ANOVA: *F* (3, 60) = 1.175, *p* = 0.327; Figure 1B). Finally, to analyze activity-dependent long-term synaptic plasticity, long-term potentiation (LTP) was induced *via* Theta burst stimulation (TBS) at the CA3-CA1 Schaffer collateral pathway. In slices derived from WT and p75^NTR^ KO, no differences could be observed in LTP, as previously reported by Rösch et al., 2005. In contrast, the APP/PS1tg slices already showed a lower LTP during the induction phase and for the entire time recorded (Two-way RM ANOVA: F (3, 76) = 10.53, *p* < 0.001; Figure 1C), which resulted in a significant difference of ~30% 55–60 min after TBS during the maintenance phase of LTP (Ordinary one-way ANOVA: F (3, 76) = 13.24, *p* < 0.0001; Figure 1D). The average potentiation reached 55–60 min after the TBS in APP/PS1tg slices was 1.242 ± 0.077, whereas WT and p75^NTR^ KO slices showed potentiation of 1.734 ± 0.076 and 1.847 ± 0.079, respectively (Figure 1D). The reduction in LTP was completely absent in APP/PS1tg x p75^NTR^ KO mice showing potentiation of 1.758 ± 0.067 which was not significantly different from the one observed in WT and p75^NTR^ KO mice. However, 55–60 min after TBS the potentiation in APP/PS1tg x p75^NTR^ KO was significantly higher than in APP/PS1tg mice *p* < 0.0001) (Figures 1C,D).

In summary, these results show that the deletion of p75^NTR^ is sufficient to completely rescue the LTP impairment observed in 10 months old APP/PS1tg mice at the CA3-CA1 Schaffer collateral pathway.

### Deletion of p75^NTR^ prevents dendritic spine loss in APP/PS1tg mice

**Figure 2:**
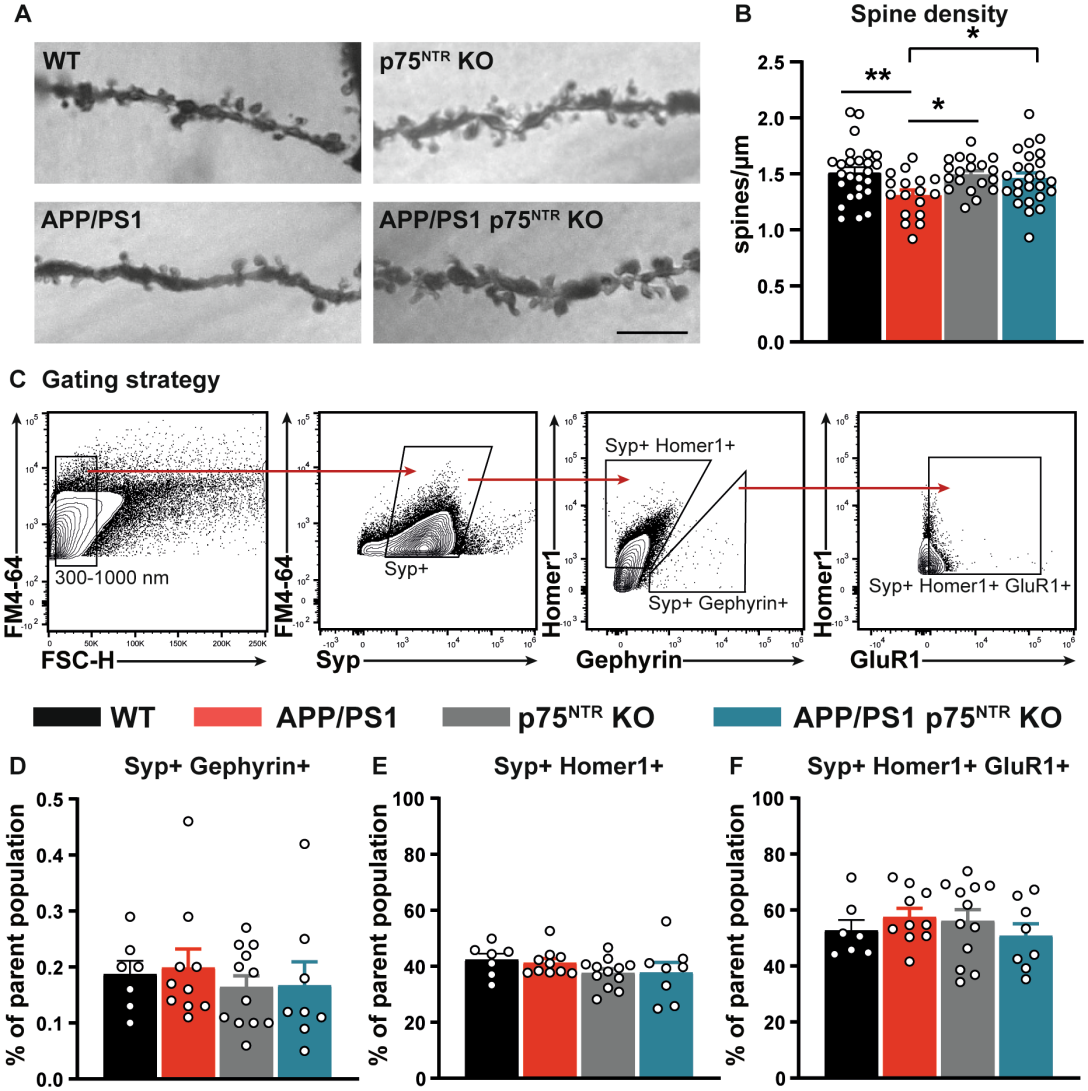
Deletion of p75^NTR^ rescued the spine pathology in APP/PS1tg mice. **(A)** Representative images of the dendritic stretches used for the analysis of dendritic spine density in WT, APP/PS1tg, p75^NTR^ KO, and APP/PS1tg x p75^NTR^ KO mice. The scale bar is 10 μm. **(B)** Spine density in APP/PS1tg mice was significantly reduced compared to controls. No reduction in spine density was observed in APP/PS1tg x p75^NTR^ KO mice. **(C)** After the selection of events between 300 and 1,000 nm, synaptosomes were gated for Synaptophysin-positive (Syp+) events. Consecutively, the events were further separated in Syp+; Homer1-positive (Homer1+) and Syp+; and Gephyrin-positive (Gephyrin+). Finally, Syp+; Homer1+ were further analyzed toward their GluR1 expression **(D-F)**. No significant differences between the experimental groups were observed for Homer1+, Gephyrin, and the combination of Homer1 + GluR1. Data are presented as mean ± SEM, ordinary one-way ANOVA **(B,E** of data, *post hoc* uncorrected Fisher’s-LSD **(B)**, and Tukey’s-multiple comparisons **(C-F)** tests were performed. ^*^p < 0.05, ^**^p < 0.01 compared to control.

The rescue of the synaptic plasticity impairment observed in the CA3-CA1 Schaffer collateral pathway of APP/PS1tg x p75^NTR^ KO hippocampus is potentially mediated either by a direct effect of p75^NTR^, acting locally to modulate excitatory synaptic transmission or indirectly by the p75^NTR^–mediated regulation of broad-scale mechanisms such as neuroinflammation. Since p75^NTR^ signaling was shown to be required for the Aβ-induced dendritic spine pathology *in vitro* (Patnaik et al., 2020), we first quantified the dendritic spine density on apical dendrites from CA1 pyramidal neurons as a proxy for excitatory synapses (Figures 2A,B). Quantification of dendritic spine density resulted in similar values between WT and p75^NTR^ KO mice, suggesting no effect of p75^NTR^ on spine density in healthy, adult mice (one-way ANOVA: *F* (3, 83) = 3.26, *p* < 0.05; WT vs. p75^NTR^ KO: *p* = 0.817). On the contrary, APP/PS1tg mice displayed a severe dendritic spine pathology with a significantly reduced spine density compared to WT mice (WT vs. APP/PS1tg: *p* < 0.01). Remarkably, this decrease was not detected in APP/PS1tg x p75^NTR^ KO mice (p75^NTR^ KO vs. APP/PS1tg x p75^NTR^ KO: *p* = 0.62; APP/PS1tg vs. APP/PS1tg x p75^NTR^ KO: p < 0.05), which indicates a crucial role of p75^NTR^ during AD on the number of dendritic spines. Recently, it was shown that the loss of glutamatergic synapses after middle artery occlusion depends on p75^NTR^ signaling, whereas the downregulation of GABAergic synapses was dependent on tropomyosin receptor kinase B (Cramer et al., 2022). We, therefore, investigated whether p75^NTR^ signaling in APP/PS1tg mice may affect glutamatergic or GABAergic synapses by taking advantage of high sensitive flow synaptometry (Düsedau et al., 2021). This approach allows the quantitative analysis of synaptic protein expression within single synaptosomes without the possible limitations due to differences in antibody penetration during immunohistochemical labeling of synaptic markers within the tissue. Quantification of hippocampal synaptosome preparations stained for synaptophysin (Syp+), Homer1 as a postsynaptic glutamatergic marker, and Gephyrin as a postsynaptic GABAergic marker revealed no significant differences in the proportion of Homer1-positive (Homer1+) or Gephyrin-positive (Gephyrin+) vesicles from the parent population between the different mouse groups (Homer1+: one-way ANOVA: *F* (3,33) = 1.212, *p* = 0.33; Gephyrin+: one-way ANOVA: F (3,33) = 0.324, *p* = 0.81 (Figures 2C,D-F). This result indicates no significant impact of p75^NTR^ signaling on the balance between excitatory and inhibitory synapses. Finally, we stained the synaptosomal population for Homer-1 and the AMPAR subunit GluR1 to investigate whether p75^NTR^ signaling alters the expression of this specific AMPAR subunit. Indeed, while the expression of the AMPAR subunits GluR2 and GluR3 were already shown to be altered in healthy p75^NTR^ KO mice, thereby influencing LTD but not LTP (Rösch et al., 2005), no data were available for GluR1. No significant differences could be detected between all groups regarding the frequency of Homer1-positive and GluR1-positive events (Homer1+ GluR1+: one-way ANOVA: *F* (3,33) = 0.61, *p* = 0.61; Figure 2F).

In summary, the rescue of the LTP phenotype in the CA3-CA1 Schaffer collateral pathway of APP/PS1tg mice is associated with a rescue of the dendritic spine pathology in APP/PS1tg x p75^NTR^ KO mice. Thus, it can be concluded that p75^NTR^ controls spine stability also under pathophysiological conditions, like in AD. Interestingly, no differences in the balance of excitatory and inhibitory synaptic markers and the expression of GluR1 were observed in synaptosomes using flow synaptometry.

### Effect of p75^NTR^ deletion in APP/PS1tg mice on microglia activation and neuroinflammation

**Figure 3:**
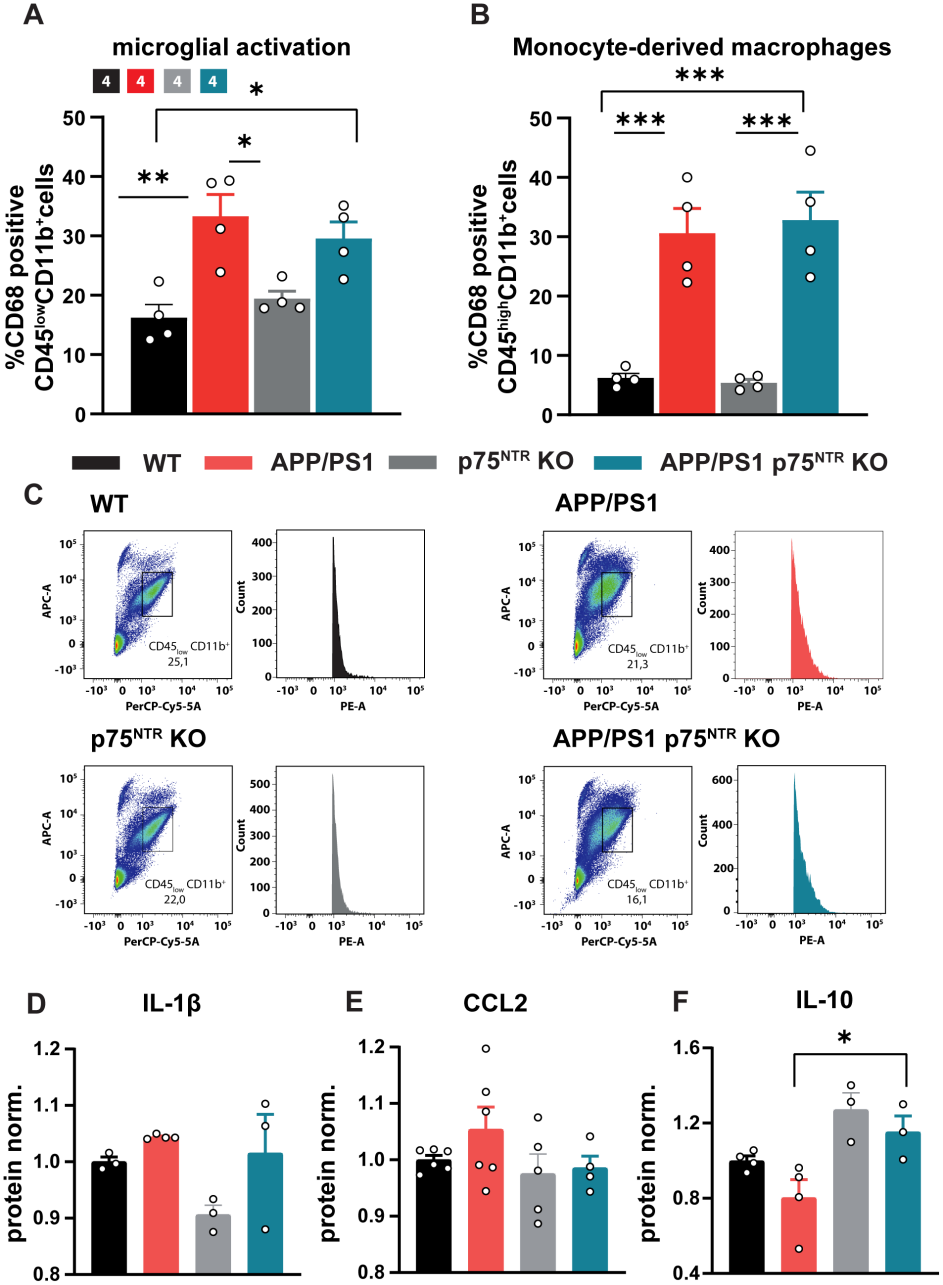
Knockout of p75^NTR^ did not ameliorate microglia and monocyte-derived-macrophage (MDM) activation in 10 months old APP/PS1tg mice. **(A,B)** The CD68 expression of CD11b^+^/CD45^low^ (microglia) and gated CD11b^+^/CD45^high^ (MDM) cells normalized to WT was significantly enhanced in APP/PS1 and APP/PS1tg x p75^NTR^ KO cells compared to controls. No reduction in the activation was observed comparing APP/PS1tg and APP/PS1tg x p75^NTR^ KO probes. **(C)** Representative plots of the gated CD11b^+^/CD45^low^ cell population and histogram of CD68 expression. Data are presented as mean ± SEM (*N* = 4) and one-way ANOVA of data, and *post hoc* Tukey multiple-comparisons test were performed. ^*^p < 0.05, ^**^p < 0.01, and ^***^p < 0.001 compared with control. **(D–F)** The graphs show respectively the relative expression levels of IL1ß, CCL2 and IL10 in the hippocampus of WT, APP/PS1tg, p75^NTR^ and APP/PS1 x p75^NTR^ KO mice.

Chronic neuroinflammation triggered by Aβo is known to be one of the drivers of the pathology and cognitive decline occurring in AD (Scheiblich et al., 2020). Indeed, the inhibition of neuroinflammation in mouse models can rescue many AD hallmarks, one of them being the LTP deficits in APP/PS1tg mice (Heneka et al., 2013; Lonnemann et al., 2020). Recent studies also indicate that chronic immune activation may suppress synaptic plasticity by promoting abnormal synaptic pruning by activated microglial cells thereby altering the excitation-inhibition balance and accelerating disease progression (Hong et al., 2016; Mottahedin et al., 2017; Andoh et al., 2019). Thus, we next assessed whether a reduction in the neuroinflammation may underlie the rescue of LTP and dendritic spine number in 10 months old APP/PS1tg x p75^NTR^ KO mice. First, the number of activated microglia cells was analyzed by flow cytometry upon labeling for the activation marker CD68. While p75^NTR^ KO mice displayed no significant differences in the proportion of activated microglia compared to WT, APP/PS1tg mice showed a significant increase in the amount of activated CD68-expressing microglia (one-way ANOVA: *F*(3,12) = 9.478, *p* < 0.01; WT: 16.23 ± 2.205, p75^NTR^ KO: 19.43 ± 1.278, APP/PS1tg: 33.33 ± 3.653; WT vs. p75^NTR^ KO: *p* = 0.82, WT vs. APP/PS1tg: *p* < 0.01). However, no significant difference could be seen between APP/PS1tg and APP/PS1tg x p75^NTR^ KO mice (APP/PS1tg x p75^NTR^ KO: 29.55 ± 2.819, APP/PS1tg vs. APP/PS1tg x p75^NTR^ KO: *p* = 0.74; Figures 3A,C), the latter having a significantly higher amount of activated microglia than WT mice (WT vs. APP/PS1tg x p75^NTR^ KO: *p* < 0.05). These results suggest no involvement of p75^NTR^ in regulating microglia activation at this stage of the disease. Since in the late stages of AD monocyte-derived macrophages (MDM) infiltrate the brain parenchyma due to deficits in the blood brain barrier and the p75^NTR^ is of known importance for macrophage activation (Williams et al., 2016; Bandoła et al., 2017; Ahn et al., 2018; Düsedau et al., 2019), we additionally assessed the activation of MDMs. The analysis revealed a strong activation of MDMs in AD mice compared to WT (one-way ANOVA: F(3,12) = 22.29, *p* < 0.0001; WT: 6.218 ± 0.748; p75^NTR^ KO: 5.393 ± 0.569; APP/PS1tg: 30.59 ± 4.167; APP/PS1tg x p75^NTR^ KO: 32.83 ± 4.696; WT vs. APP/PS1tg: *p* < 0.001, WT vs. APP/PS1tg x p75^NTR^ KO: *p* < 0.001, p75^NTR^ KO vs. APP/PS1tg x p75^NTR^ KO: *p* > 0.001). Knockout of p75^NTR^ did not influence the activation level of the monocyte population (WT vs. p75^NTR^ KO: *p* > 0.99; Figure 3B). To further investigate the degree of neuroinflammation, cytokine expression was analyzed in the hippocampus using an enzyme-linked immunoabsorbent assay (ELISA). In agreement with the higher microglia activation indicated by the flow cytometry results, APP/PS1tg and APP/PS1tg x p75^NTR^ KO animals displayed a trend toward a higher IL-1β expression compared to controls (Figure 3D). Similarly, for the chemokine CCL2, a trend for a higher expression was seen in APP/PS1tg animals compared to all other experimental groups, albeit not significantly (Figure 3E). Next, the expression of IL-10, an anti-inflammatory cytokine associated with synapse formation (Lim et al., 2013; Chen et al., 2016), facilitation of synaptic transmission (Nenov et al., 2019), and restoration of LPS-induced alteration in synaptic plasticity (Lenz et al., 2020), was analyzed. The results show significant changes in IL-10 expression levels between the experimental groups (one-way ANOVA: *F*(3,10) = 6.809, *p* < 0.01) (Figure 3F). While the expression of IL-10 was slightly increased in p75^NTR^ KO and APP/PS1tg x p75^NTR^ KO and slightly decreased in APP/PS1tg mice compared to the respective controls, its expression in APP/PS1tg was significantly lower compared to APP/PS1 x p75^NTR^ KO mice (APP/PS1tg vs. APP/PS1tg x p75^NTR^ KO: *p* < 0.05). These results suggest that p75^NTR^ could be involved in specifically regulating cytokine signaling pathways associated with the modulation of synaptic density and plasticity.

**Figure 4:**
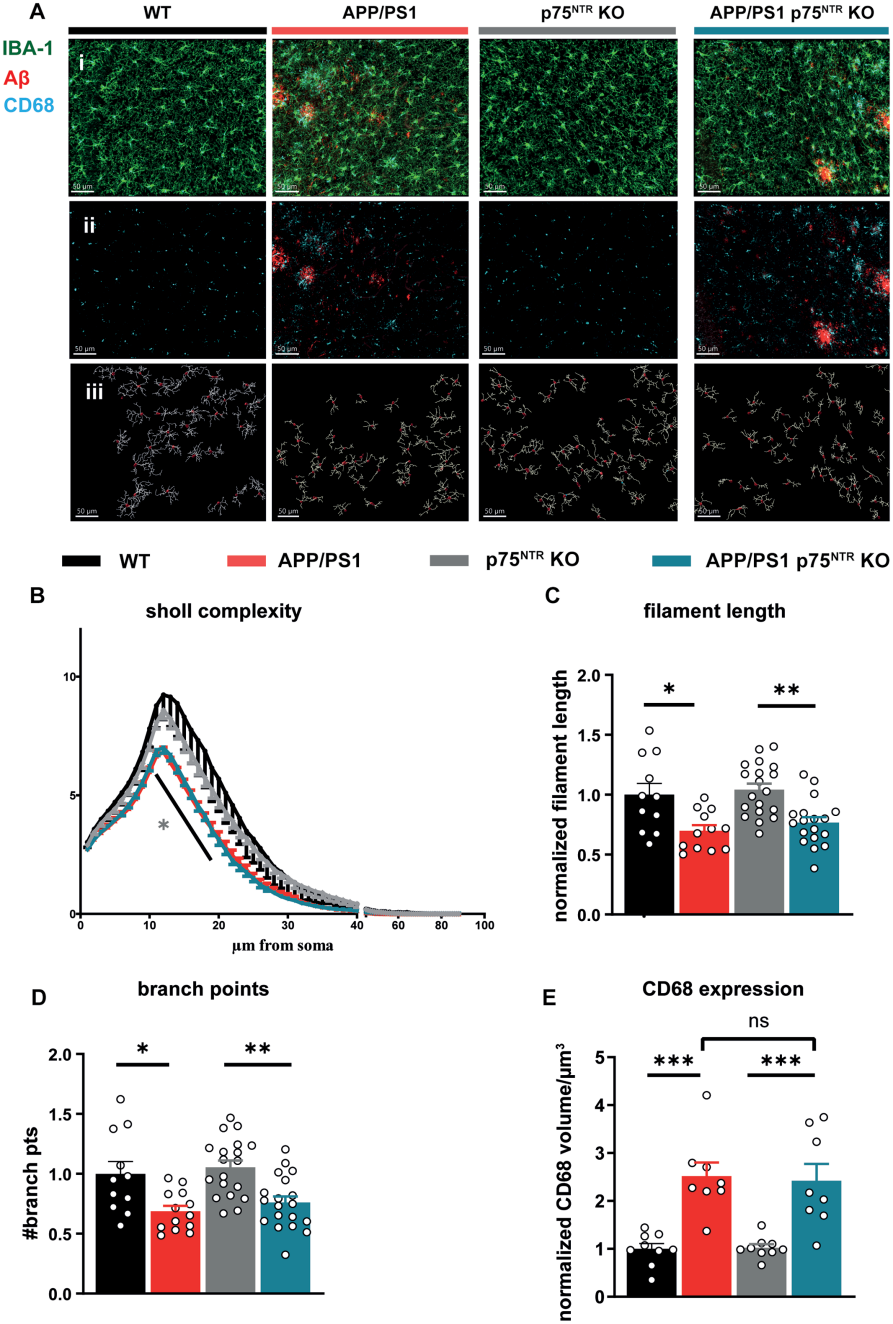
Deletion of the p75^NTR^ did not influence the morphology of microglia in 10 months old APP/PS1tg mice. **(A)** Immuno staining of Iba1 (green), CD68 (cyan), and Amyloid-ß (red) in the CA1 area of the hippocampus from mice of all experimental groups (i + ii). Based on the staining, 3D filament models were generated, and the Sholl analysis was performed in IMARIS® (iii), scale bar = 50 μm. **(B)** Microglial complexity was reduced in APP/PS1tg and APP/PS1tg x p75^NTR^ KO mice but not in controls. (grey^*^ comparison to p75^NTR^ KO). **(C)** Total filament length. **(D)** Branch points of Iba1+ cells were equally and significantly decreased in APP/PS1tg and APP/PS1tg x p75^NTR^ KO mice compared to controls. Deletion of p75^NTR^ KO did not influence this result. **(E)** Expression of CD68 within microglia (shown as CD68+ volume) was significantly increased in APP/PS1tg and APP/PS1tg x p75^NTR^ KO mice but not in controls. However, deletion of p75^NTR^ did not result in significant differences in CD68 expression in APP/PS1tg mice (N = WT:4; APP/PS1tg: 4; p75^NTR^ KO: 7; APP/PS1tg x p75^NTR^ KO: 7 animals per group n (number of ROIs) = 3 per animal). Data are presented as mean ± SEM. Two-way repeated measures ANOVA in **(B)**, ordinary one-way ANOVA of data **(C-E),** and *post hoc* Tukey multiple-comparisons test were performed. ^*^p < 0.05, ^**^p < 0.01, and ^***^p < 0.001 compared with control.

Finally, immunofluorescence was used to label the microglia markers, Iba1 and CD68, in order to analyze microglia activation *in situ* within the CA1 region of the hippocampus. The typical changes occurring in the morphology of microglia upon activation, characterized by the retraction of their processes and a reduced complexity were analyzed (Beynon and Walker, 2012; Verdonk et al., 2016; Franco-Bocanegra et al., 2021). Therefore, microglial filament models were generated based on the Iba1 staining and subsequently a Sholl analysis was performed using the IMARIS® software (Figure 4A). Analysis of complexity revealed a significantly reduced complexity of microglia in APP/PS1tg and APP/PS1tg x p75^NTR^ KO mice compared to controls (two-way RM ANOVA: *F*(3,27) = 9.752, *p* < 0.001; Figure 4B). This observation was also true for filament length (one-way ANOVA: *F*(3,56) = 8.608, *p* < 0.0001; WT vs. APP/PS1tg: p < 0.05, p75^NTR^ KO vs. APP/PS1tg x p75^NTR^KO: p < 0.01; Figure 4) and numbers of branching points (one-way ANOVA: *F*(3,58) = 8.429, p < 0.0001; WT vs. APP/PS1tg: p < 0.05, p75^NTR^ KO vs. APP/PS1tg x p75^NTR^KO: p < 0.05; Figures 4B–D). The complexity, filament length, and the number of branching points did not differ between WT and p75^NTR^ KO as well as between APP/PS1tg and APP/PS1tg x p75^NTR^ KO-derived microglia Figures 4A-D). Assessing the region-specific CD68 expression confirmed the quantification using flow cytometry and showed no significant difference in the CD68 expression within microglia between APP/PS1tg and APP/PS1tg x p75^NTR^ KO; however, both the levels of CD68 expression were significantly higher compared to those in WT and p75^NTR^ KO mice, respectively (WT: 1 ± 0.112; p75^NTR^ KO: 1.023 ± 0.073; APP/PS1tg: 2.517 ± 0.285, to WT *p* < 0.001; APP/PS1tg x p75^NTR^ KO: 2.425 ± 0.349 to WT *p* < 0.001, to p75^NTR^ KO *p* < 0.001; Figure 4E). Of note is that in 10 month old APP/PS1tg, the area of amyloid-β plaques present in the hippocampus was higher, albeit not significantly than in APP/PS1tg x p75^NTR^ KO mice (Supplementary Figure S1). This difference became significant in 18 month old mice due to an increase in plaque area observed in APP/PS1tg but not in APP/PS1tg x p75^NTR^ KO mice (Supplementary Figure S1).

Taken together, these data confirm the previously shown strong inflammation in old APP/PS1tg mice (Heneka et al., 2013; Lonnemann et al., 2020). Moreover, the results show that the deletion of p75^NTR^ does not influence microglia morphology and their activation in healthy mice and does not prevent it in APP/PS1tg mice. Thus, it is unlikely that a reduction in neuroinflammation is the mechanism by which a loss-of-function for p75^NTR^ rescues the LTP and dendritic spine deficits in the APP/PS1 transgenic mice. However, the elevated IL-10 expression in p75^NTR^ KO and APP/PS1tg x p75^NTR^ KO animals indicates a possible involvement of p75^NTR^ in the modulation of specific cytokine pathways suggested to modulate synaptic transmission.

### A p75^NTR^ deletion did not prevent the deficits in spatial learning and memory in APP/PS1 transgenic mice

**Figure 5:**
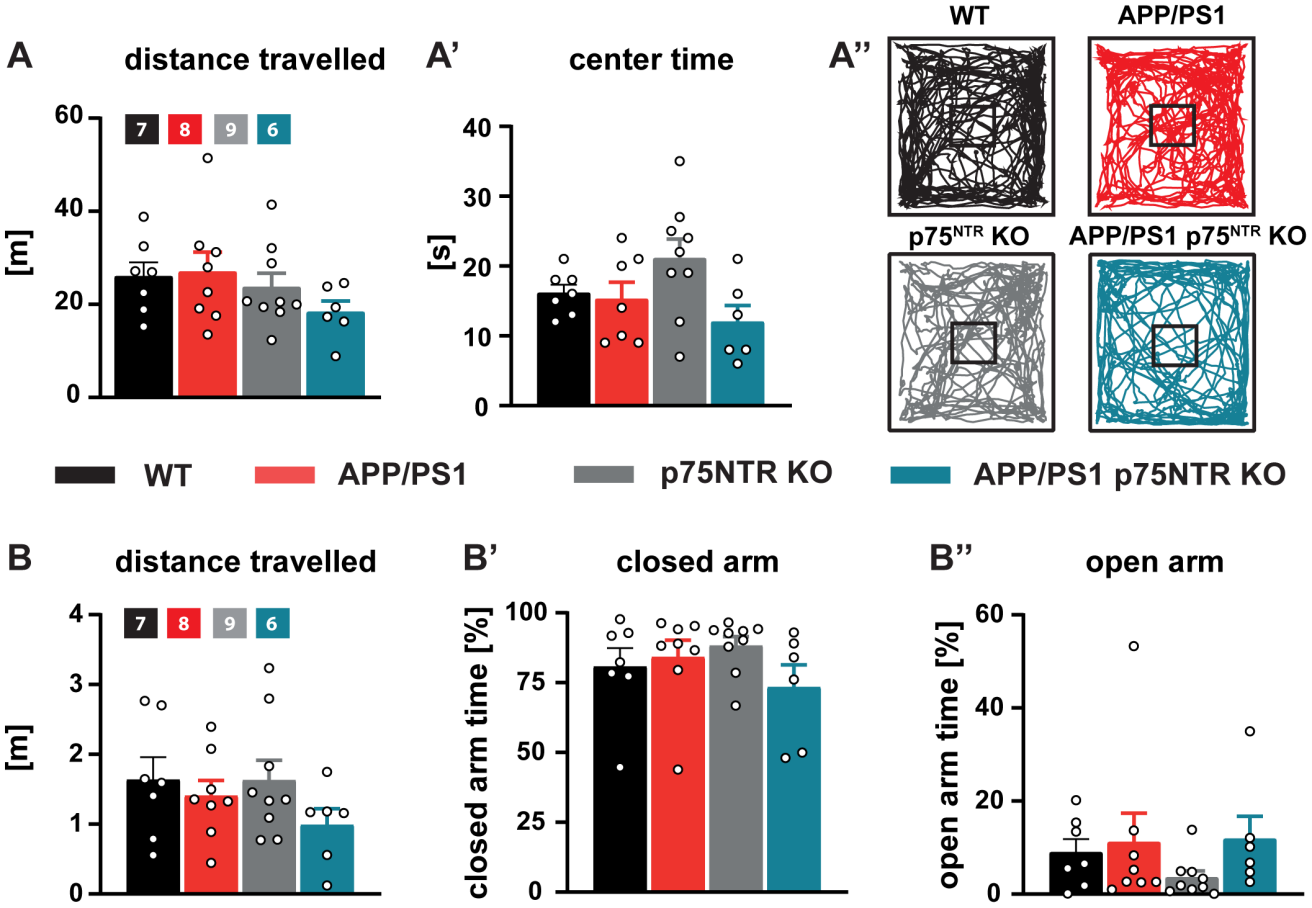
General locomotion, willingness to explore, and anxiety behavior were unaffected in APP/PS1tg and APP/PS1tg x p75^NTR^ KO mice. **(A, A’)** The total distance traveled and time spent in the center area of the open field arena were similar in all groups**. (A”)** Representative track plots of the movement of WT, APP/PS1tg, p75^NTR^ KO, and APP/PS1tg x p75^NTR^ KO animals in the open field arena. **(B-B”)** The total distance traveled, and time spent in the closed and open arms of the elevated plus maze did not differ between the experimental groups. Data are presented as mean ± SEM (*N* = 6-9), ordinary one-way ANOVA of data and *post hoc* Tukey multiple-comparisons test were performed.

**Figure 6:**
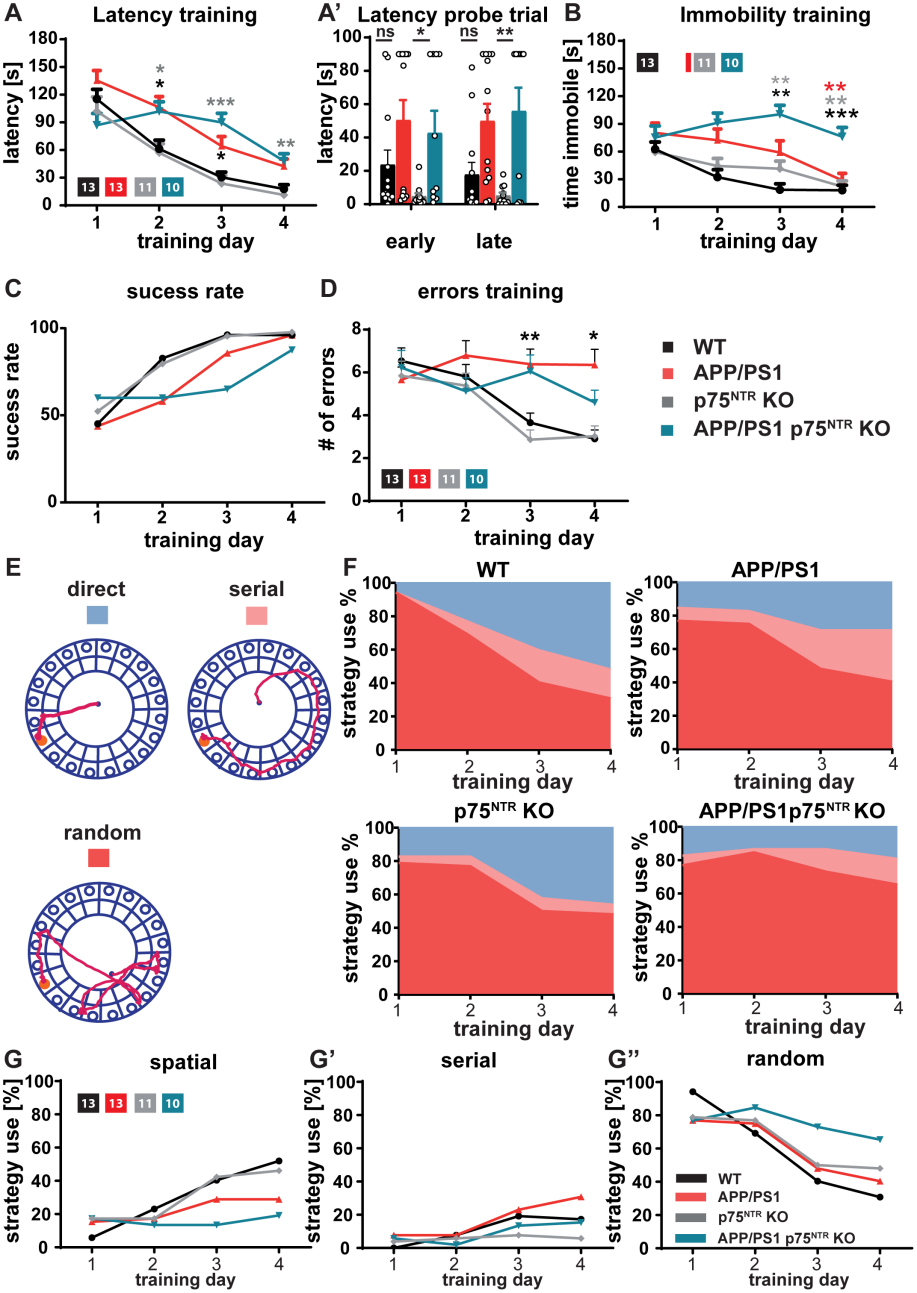
Deficits in spatial learning and memory in APP/PS1tg mice were not rescued by p75^NTR^ deletion. Spatial memory was assessed using the Barnes maze task **(A-A’).** The latency to locate the target hole in the Barnes maze during the training session and probe trials were elevated in APP/PS1tg and APP/PS1tg x p75^NTR^ KO animals compared to controls. Deletion of p75^NTR^ did not rescue the deficit. **(B)** The time spent immobile during training in the Barnes maze was similar in WT, APP/PS1tg, and p75^NTR^ KO mice; however, APP/PS1tg x p75^NTR^ KO mice displayed significantly higher immobility. **(C)** APP/PS1tg mice were less successful in solving the task by escaping the maze through the escape hole compared to controls (success rate). A p75^NTR^ KO did not improve the success rate. (**D**) The APP/PS1tg mice showed a significantly higher number of errors in locating the target hole compared to controls; this defect was not rescued by p75^NTR^ deletion. **(E-G”)** The analysis of the search strategies used to solve the task revealed no significant improvement in the spatial deficits displayed by APP/PS1tg mice after p75^NTR^ deletion. APP/PS1tg and APP/PS1tg x p75^NTR^ KO animals used less hippocampus-dependent and slightly more hippocampus-independent strategies. **(E)** Representative track plots for direct (hippocampus-dependent), serial, and random (hippocampus-independent) search strategies used to solve the Barnes maze task. **(F)** Area plot reporting the percentage of strategy use per experimental group. **(G-G”)** Graphs showing the direct comparison of experimental groups with regard to the search strategies. Two way repeated measures ANOVA and *post hoc* Tukey multiple-comparisons test were performed. ^*^p < 0.05, ^**^p < 0.01, and ^***^p < 0.001 (grey^*^ comparison to p75^NTR^ KO, black* comparison to WT, red* comparison to APP/PS1tg).

The hippocampus and the LTP at the CA3-CA1 Schaffer collateral pathway have been shown to play a central role in the formation and maintenance of long-term spatial memory (Maren and Baudry, 1995; Lynch, 2004), for a review see Korte and Schmitz, 2016. Therefore, we next investigated to which extent, despite the unaltered neuroinflammation, the rescue of the LTP at the CA3-CA1 Schaffer collateral pathway and the dendritic spine density by the deletion of p75^NTR^ might prevent the cognitive decline observed in APP/PS1tg mice. Indeed, among the most prominent hallmarks of AD models is the progressive cognitive decline as shown in different mouse models using the gradual deterioration of spatial memory (Hsiao et al., 1996; Sturchler-Pierrat et al., 1997; Serneels et al., 2009). First, the mice were analyzed for their explorative and anxiety-related behavior using the Open Field Test and the Elevated Plus-Maze to ensure comparability between the mouse groups. Analysis of the Open Field Test revealed no difference in distance traveled or time spent in the center versus the periphery of the arena between WT and p75^NTR^ KO as well as no significant impairment in APP/PS1tg and APP/PS1tg x p75^NTR^ KO mice (Figures 5A-A”). Analysis of the traces in the Elevated Plus-Maze also showed no significant difference in distance traveled and time spent in the closed versus the open arm time (Figures 5B-B”) indicating no differences in the levels of anxiety between the different mouse genotypes. Spatial learning and memory formation were assessed using the Barnes Maze (Barnes, 1979; Harrison et al., 2006). This behavioral test was already used for the p75^NTR^ KO mice (Barrett et al., 2010; Murphy et al., 2015) and was preferred over the Morris water maze, since in our previous behavioral analysis they showed significantly altered swimming speed compared with WT mice, possibly influencing the results. The performance in the maze was evaluated by analyzing the trajectory of the mice until they visited the escape hole for the first time. For all groups, we observed significant learning (one-way RM ANOVA WT: *F*_(3,36)_ = 47.71, *p* < 0.0001; p75^NTR^ KO: F_(3,36)_ = 18.30, p < 0.0001 APP/PS1tg: F_(3,36)_ = 24.37, p < 0.0001; APP/PS1tg x p75^NTR^ KO: *F*_(3,27)_ = 6.115, *p* < 0.01) shown by the progressive reduction of the latency to reach the correct hole over the course of 4 training days (Figure 6A). However, compared to WT mice, APP/PS1tg mice displayed a higher latency over the entire training time, which became statistically significant on days 2 and 3, reflecting the impairment of spatial memory in APP/PS1tg mice (two-way RM ANOVA: *F*(3,43) = 8.415, *p* < 0.001; WT vs. APP/PS1tg: day2, *p* < 0.05; day3, p < 0.05; Figure 6A). A similar phenotype was observed in APP/PS1tg x p75^NTR^ KO mice showing significantly higher latency than WT on days 2, 3, and 4 (WT vs. APP/PS1tg x p75^NTR^ KO: day2, p < 0.05; day3, p < 0.001; day4, p < 0.05; Figure 6A). A direct comparison of APP/PS1tg x p75^NTR^ KO with APP/PS1tg mice however, showed no significant difference for all training days (Figure 6A). This was also true for the results of the probe trials indicating no rescue of spatial memory due to the deletion of p75^NTR^ (Figure 6A’). Indeed, for both short- and long-term probe trials, the latency was higher, albeit not significant in APP/PS1tg compared to WT mice but significantly higher in APP/PS1tg x p75^NTR^ KO compared to p75^NTR^ KO mice (two-way RM ANOVA: F(3,43) = 6.74, p < 0.001; p75^NTR^ KO vs. APP/PS1tg x p75^NTR^ KO: short-term: *p* = 0.05; long-term, p < 0.05; Figure 6B). No significant difference was observed between WT and p75^NTR^ KO mice (WT vs. p75^NTR^ KO: short-term: *p* = 0.51, long-term: *p* = 0.79; Figure 6A’). It is important to note that the time of immobility for the APP/PS1tg x p75^NTR^ KO was significantly increasing throughout the training possibly affecting their latency. Indeed, on day 4, APP/PS1tg x p75^NTR^ KO mice spent significantly more time immobile than all other groups (two-way RM ANOVA: F(3,43) = 11.70, p < 0.0001; vs. WT p < 0.001, vs. APP/PS1tg p < 0.05, vs. p75^NTR^ KO *p* = 0.001; APP/PS1tg x p75^NTR^ KO: 76.09 s ± 10.08 s, WT: 18.14 s ± 5.48 s, p75^NTR^ KO: 22.08 s ± 6.17 s; APP/PS1tg 28.89 s ± 7.5 s; Figure 6B). Next, time-independent measures of successfully solving the spatial task were assessed including success rate, number of errors, and search strategies used. The success rate describes how frequently the mice solved the task. While the success rate progressively increased with the training equally in all groups, APP/PS1tg and APP/PS1tg x p75^NTR^ KO mice showed a slower increase in success rate, especially on days 2 to 4 of the training, albeit not significantly. While, APP/PS1tg mice performed equally to WT and p75^NTR^ KO mice on the last day (WT: 96.07 %, APPPS1tg: 96%, p75^NTR^: 97.72%), APP/PS1tg x p75^NTR^ KO were slightly worse (APP/PS1tg x p75^NTR^ KO: 87.5%; Figure 6C) indicating that deletion of p75^NTR^ does not rescue the deficit of spatial memory in APP/PS1tg mice but rather results in a slightly stronger impairment. Next, we analyzed the number of errors. WT and p75^NTR^ KO mice showed a progressive decline in the number of errors for each consecutive training day (one-way RM ANOVA WT: *F*(3, 36) = 6.394, p < 0.01; p75^NTR^ KO: *F*(3, 30) = 3.677, p < 0.05; Figure 6D). On the contrary, APP/PS1tg and APP/PS1tg x p75^NTR^ KO mice showed no significant decrease in the number of errors over the training days (one-way RM ANOVA APP/PS1tg: F(3, 36) =0.458, *p* = 0.713; APP/PS1tg x p75^NTR^ KO: *F*(3, 27) =1.036, *p* = 0.391; Figure 6D**).** Moreover, APP/PS1tg mice made significantly more mistakes than the healthy mice on day 3, whereas APP/PS1tg x p75^NTR^ KO mice showed only a trend of increased mistakes, possibly due to less movement (two-way RM ANOVA: F(3,43) = 3.28, *p* < 0.05; APP/PS1tg vs. WT: *p* < 0.05, APP/PS1tg vs. p75^NTR^ *p* = 0.001; APP/PS1tg x p75^NTR^ vs. WT: *p* = 0.17, APP/PS1tg x p75^NTR^ KO vs. p75^NTR^ KO *p* = 0.058, WT: 3.65 ± 0.45, p75^NTR^ KO: 2.86 ± =0.45, APP/PS1tg: 6.38 ± 0.7, APP/PS1tg x p75^NTR^ KO: 6.05 ± 0.77; Figure 6D). In the final step, we assessed the specific role of the hippocampus in solving this spatial task and analyzed the search strategies the mice used to solve the task by categorizing them into spatial (hippocampus-dependent), serial, and random (hippocampus-independent) (Figures 6E-G”). All groups displayed similar search strategy patterns in the first 2 days of training characterized by the prevalent use of hippocampus-independent strategies (Figures 6G-G”). Over the last 2 days, however, WT and p75^NTR^ KO mice equally increased their use of spatial, hippocampus-dependent strategies (day 4, WT: 51.92%, p75^NTR^ KO: 46.15%). In contrast, APP/PS1tg mice showed only a mild increase in spatial strategies (day 4, 28.85%) and a more prominent increase in serial strategies, indicating a deficit specifically in hippocampus-dependent learning. APP/PS1tg x p75^NTR^ KO mice used less spatial (day 4, 19.23%) and serial search strategies and displayed the highest use of random strategies of all groups thereby performing slightly worse than the APP/PS1tg mice (Figures 6G-G”).

Taken together, the behavioral analysis shows no rescue of the spatial memory decline observed in 10 months old APP/PS1tg mice upon deletion of p75^NTR^. Success rate and strategy analysis even indicated a slightly stronger impairment upon p75^NTR^ knockout. In addition, we show that the knockout of p75^NTR^ in healthy mice does not influence their ability for spatial learning and memory.

## Discussion

This study addresses the role of the p75^NTR^ receptor in mediating the primary symptoms and pathological alterations of Alzheimer’s disease (AD). The *in vitro* evidence so far suggested that the p75^NTR^ is mediating the cellular and synaptic toxicity of Aβ oligomers (Aβo) (Sotthibundhu et al., 2008; Patnaik et al., 2020). On the other hand, the analysis performed upon its deletion *in vivo* in different mouse models of AD (Wang et al., 2011; Murphy et al., 2015; Jian et al., 2016; Qian et al., 2018) did not unequivocally confirm the role of p75^NTR^ in the pathogenesis and progression of the disease. Thus, here we analyzed the consequences of complete deletion of p75^NTR^ in a well-studied AD mouse model by crossing the APP/PS1 transgenic (APP/PS1tg) to the p75^NTRexonIV^ knockout (p75^NTR^KO) mice. Our findings suggest very complex actions of p75^NTR^ in mediating the pathogenesis of AD, involving a direct role in specifically mediating the synaptic pathology but no other aspects of the pathogenesis. The results of this study show that, while the complete deletion of p75^NTR^ rescues the impaired activity-dependent synaptic plasticity and the loss of dendritic spines typical of 10 months old APP/PS1tg mice, it does not suppress the chronic neuroinflammation and microglial activation. Moreover, the cognitive decline typical of the advanced phase of the disease, as observed by analyzing processes of hippocampus-dependent spatial learning is not prevented in APP/PS1tg x p75^NTR^ KO mice.

The complete rescue of the dendritic spine loss and the impairment in long-term potentiation (LTP) observed in this study is in agreement with previous observations proving a significant impact of p75^NTR^ signaling in regulating synapse stability and plasticity. Indeed, p75^NTR^ is involved in the regulation of dendritic complexity as well as dendritic spine number and morphology (Zagrebelsky, 2005). Furthermore, the binding of Aβo to p75^NTR^ was shown to activate the downstream RhoA/ROCK/actin pathway resulting in dendritic spine loss and enhanced Aβ localization at synapses, thereby potentially promoting the binding of Aβ-oligomers to other cellular receptors (Patnaik et al., 2020). In addition, p75^NTR^ signaling is crucial for some manifestations of activity-dependent synaptic plasticity, e.g., the ablation of p75^NTR^ results in the impairment in the maintenance of long-term depression (LTD) possibly by the regulation of specific α-amino-3-hydroxy-5-methyl-4-isoxazolepropionic acid (AMPA) and N-methyl-D-aspartate receptor **(**NMDA) receptor subunits (Rösch et al., 2005; Woo et al., 2005). On the contrary, most studies did not report any effect on LTP of either blocking p75^NTR^ signaling with antibodies (Xu et al., 2000) or by its genetic deletion both in exonIV and exonIII p75^NTR^KO mice (Rösch et al., 2005; Woo et al., 2005). The one exception is the report of Barrett et al. (2010) showing an enhanced LTP at the Shaffer collateral pathways of p75^NTRexonIII^ KO mice associated with improved spatial learning (Barrett et al., 2010). Interestingly, the application of a small molecule p75^NTR^ ligand rescues the Aβ-induced impairment in LTP in acute hippocampal slices possibly by preventing the inactivation of plasticity-related CREB (Yang et al., 2008). Moreover, while the deletion of p75^NTR^ did not affect LTP under control conditions, it rescued the deficits in late-LTP induced under conditions of sleep deprivation (SD) by preventing SD-mediated effects on hippocampal cAMP–CREB-BDNF, cAMP-PKA-LIMK1-cofilin, and RhoA-ROCK2 pathways (Wong et al., 2019). Taken together, these studies suggest a direct regulatory effect of p75^NTR^ on specific plasticity-related signaling pathways possibly underlying the rescue of LTP in APP/PS1tg x p75^NTR^KO mice.

Previous studies reported a significant amelioration of the cognitive impairment in different AD mouse models upon the reduction in p75^NTR^ expression level by knocking it out (Murphy et al., 2015; Jian et al., 2016; Qian et al., 2018) or by application of small molecule ligands modulating p75^NTR^ function (Nguyen et al., 2014; Simmons et al., 2014). Indeed, these approaches prevented or significantly ameliorated the defects in spatial learning and memory formation and in cued fear conditioning typically observed in APP/PS1tg (Murphy et al., 2015) or Tg2576 (Jian et al., 2016) mice. These observations are different from our results showing no benefit of a p75^NTR^ deletion on the impairment in spatial learning in 10 months old APP/PS1tg mice. This difference in experimental outcomes may depend upon the use of different approaches to delete p75^NTR^ and the different genetic backgrounds of the mice used. Indeed, some of the previous studies (Murphy et al., 2015; Jian et al., 2016) used the p75^NTRexonIII^ knockout mouse (Lee et al., 1992). While in this mouse model, the expression of the full-length receptor is completely prevented, the short protein isoform of p75^NTR^ is still expressed (von Schack et al., 2001). Whereas targeting the exon IV of p75^NTR^, as done in this study, results in the complete ablation of both naturally occurring p75^NTR^ isoforms (von Schack et al., 2001). Indeed, mice homozygous for the exon IV null mutation show a significantly more pronounced phenotype when compared to the exon III p75^NTR^ KO mice (von Schack et al., 2001). In this context, it should be noted that in the exon IV p75^NTR^ KO, the expression of a short p75^NTR^ fragment was reported and shown to activate pro-apoptotic pathways when over-expressed in PC12 cells (Paul et al., 2004). While the activation of pro-apoptotic pathways was never shown *in vivo* in the exon IV p75^NTR^ KO, an effect of this fragment in the APP/PS1tg x p75^NTR^ KO mouse used here cannot be completely ruled out. Furthermore, most of the previous analyses only investigated partial deletions of the p75^NTR^ using hemizygous mice (Murphy et al., 2015; Jian et al., 2016) or conditional deletions of p75^NTR^ from specific cell populations e.g. cholinergic neuron (Qian et al., 2018). Interestingly, the only earlier study analyzing the consequence of a homozygous deletion of the p75^NTR^ exon III in APP/PS1tg mice failed to report any amelioration of the spatial memory deficits in the Morris water maze (Wang et al., 2011) despite a reduction in Aβ production. The latter study supports our conclusions that the full deletion of p75^NTR^ does not rescue the cognitive impairment in AD mice possibly indicating a dose-dependent effect. Moreover, a recent study showed that while crossing 5xFAD mice with p75^NTR^ knock-in mice lacking the death domain or conserved transmembrane Cys^259^ completely rescued the impairment in long-term synaptic plasticity and memory, crossing them with the p75^NTRexonIII^ KO did not (Yi et al., 2021) suggest an important signaling-independent neuroprotective function of p75^NTR^. It is also noteworthy that the role of p75^NTR^ in spatial learning is still controversial. While Peterson et al. (1999) reported an impaired performance of p75^NTR^KO mice in the Morris water maze, others found a significantly improved spatial learning (Peterson et al., 1999; Greferath et al., 2000; Murphy et al., 2015) underlying the difficulties in determining the activity of p75^NTR^ in this context. Together these observations suggest that the role of p75^NTR^ in this context is extremely complex possibly also due to its two-faced role in the pathogenesis of AD and due to the fact that in gene ablation studies p75^NTR^ is also missing during the life time of the animals, in particular during development, which could affect pruning of, e.g., cholinergic fibers into the hippocampus. While full length p75^NTR^ mediates Aβ generation and neurotoxicity, the cleaved p75^NTR^ extracellular domain (p75^ECD^) is a physiological factor protective against Aβ generation, neurotoxicity, and deposition in the brain. Indeed, p75^NTR^ directly binds Aβ (Yaar et al., 1997, 2008; Fombonne et al., 2009) and mediates the Aβ-induced cell death (Troy et al., 2002; Costantini et al., 2005; Sotthibundhu et al., 2008) and synapse pathology (Calabrese et al., 2007; Zempel and Mandelkow, 2012; Patnaik et al., 2020). On the other hand, the expression of p75^NTR^ protects cultivated hippocampal neurons against Aβ-induced toxicity (Zhang et al., 2003; Costantini et al., 2005; Blöchl and Blöchl, 2007). Moreover, p75^NTR^ mediates the endocytosis and degradation of Aβ peptides (Ovsepian et al., 2014) and p75^ECD^ promotes Aβ clearance (Wang et al., 2011; Zhou and Wang, 2011; Yao et al., 2015). Interestingly, p75^ECD^ levels decrease in AD patients and mouse models resulting in a shift in the p75^ECD^/p75^NTR^ balance toward more toxicity-mediating full-length p75^NTR^ (Yao et al., 2015). Restoring the physiological levels of p75^ECD^ alleviates AD pathologies and improves learning and memory in both the early and later phases of AD (Yao et al., 2015). Due to the complete deletion of p75^NTR^, its extracellular domain is missing in the APP/PS1tg x p75^NTRexonIV^ KO mice studied here possibly contributing to the lack of rescue of the cognitive impairments. Remarkably, in spite of the complete rescue in APP/PS1tg x p75^NTR^ KO mice of the alterations in synaptic structure and plasticity, no benefits could be observed regarding the behavioral phenotype. One possible explanation is that the strong microglial activation and neuroinflammation still present in APP/PS1tg x p75^NTR^ KO mice underlies the lack of rescue of the deficits in spatial memory. While several studies associate severe neuroinflammation with network defects and synapse loss (Heneka et al., 2013; Ising et al., 2019; Roy et al., 2020; Manabe et al., 2021), these two processes seem to be uncoupled in APP/PS1tg x p75^NTR^ KO animals. A possible explanation lays in the elevated levels of the anti-inflammatory interleukin-10 (IL-10) in APP/PS1tg x p75^NTR^ KO mice. *In vitro* studies showed a dose-dependent effect of IL-10 in regulating synaptic transmission and in facilitating LTP (Suryanarayanan et al., 2016; Nenov et al., 2019; Patel et al., 2021). Furthermore, IL-10 exerts neuroprotective and recovery-promoting effects at the network and synaptic level (Chen et al., 2016) with increased IL-10 expression levels being associated with a rescue of the neuronal architecture in p75^NTR^ KO mice infected with *T. gondii* (Düsedau et al., 2019). On the other hand, the increase in IL-10 production seems not to be sufficient to counteract the possible negative effects of microglial activation, including the increased secretion of pro-inflammatory cytokines on learning and memory processes. However, whether the lack of p75^NTR^ may promote a neuroprotective activity of microglia remains still open.

In summary, we found that a complete deletion of p75^NTR^ rescues the synaptic impairment in APP/PS1tg mice, but does not prevent microglia activation and neuroinflammation and does not slow down the cognitive impairment typical of the advanced phases of the disease. Taken together, our results contribute to interpreting the pathogenesis of Alzheimer’s disease and indicate a complex role of p75^NTR^ possibly due to its multi-faceted activities in regulating synaptic transmission, Aβ-induced toxicity, and neuroinflammation.

## Data availability statement

The original contributions presented in the study are included in the article/Supplementary material, further inquiries can be directed to the corresponding author.

## Ethics statement

The animal study was reviewed and approved by the animal welfare representative of the TU Braunschweig and the LAVES (Oldenburg, Germany, Az. §4 (02.05) TSchB TU BS and Az.33.19-42502-04-20/3498).

## Author contributions

HD, MK, and MZ: conceptualization. HD and HPD: formal analysis. HD, HPD, and SH: investigation. IRD, MK, and MZ: resources. HD and MZ: writing—original draft preparation. HD, SH, IRD, MK, and MZ: writing—review and editing. MK and MZ: supervision. MK and IRD: funding acquisition. All authors contributed to the article and approved the submitted version.

## Funding

SFB 854 project A25 to IRD and MK and RTG 2413 SynAGE to IRD. We acknowledge the support by the Open Access Publication Funds of Technische Universität Braunschweig.

## Conflict of interest

The authors declare that the research was conducted in the absence of any commercial or financial relationships that could be construed as a potential conflict of interest.

## Publisher’s note

All claims expressed in this article are solely those of the authors and do not necessarily represent those of their affiliated organizations, or those of the publisher, the editors and the reviewers. Any product that may be evaluated in this article, or claim that may be made by its manufacturer, is not guaranteed or endorsed by the publisher.
