# Supplementary figures and images for: Deletion of p75NTR rescues the synaptic but not the inflammatory status in the brain of a mouse model for Alzheimer’s disease
Source: Front Mol Neurosci. 2023 May 5;16:1163087. doi: 10.3389/fnmol.2023.1163087 (PMC10198655; doi:10.3389/fnmol.2023.1163087)

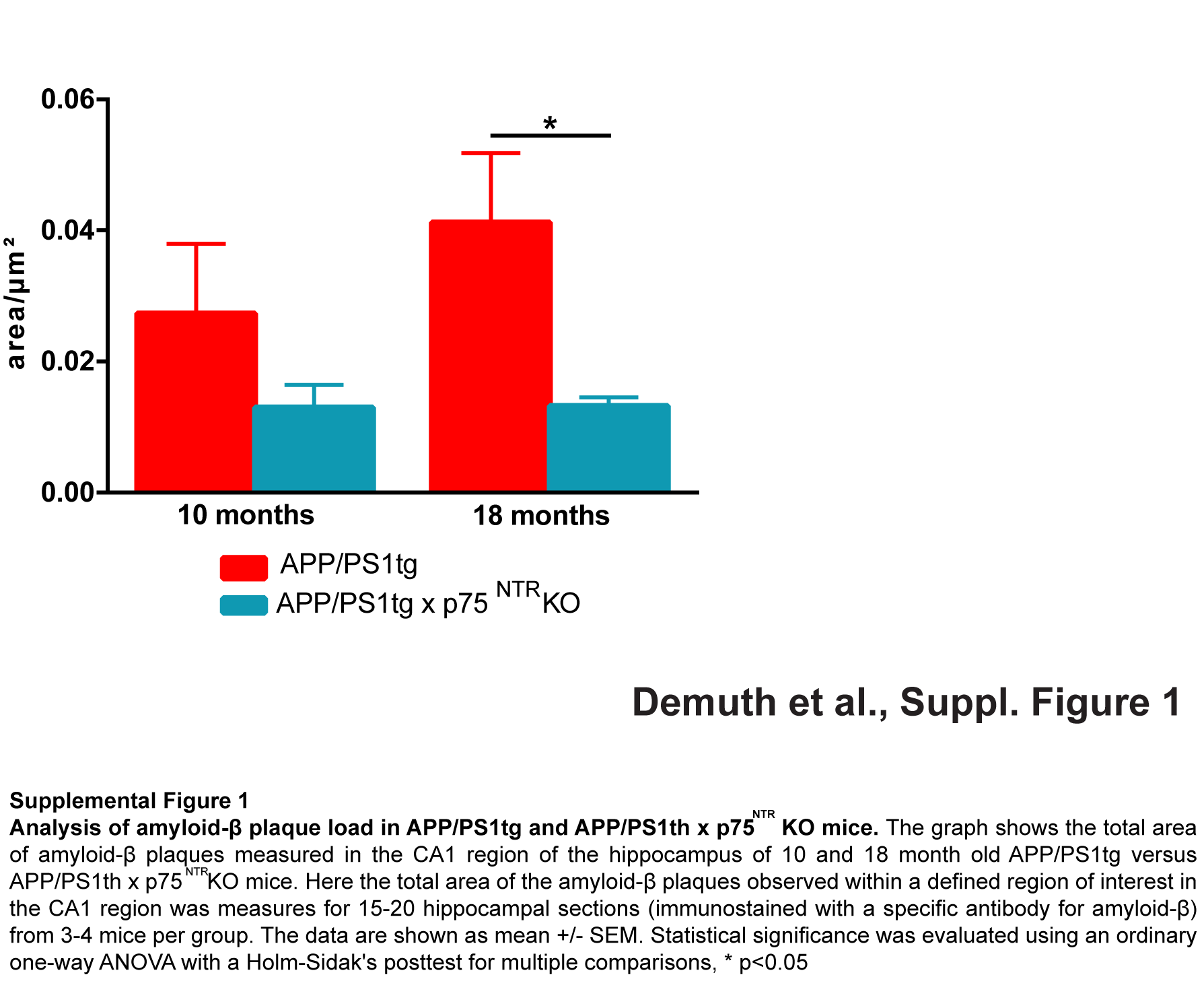

Supplement: Supplementary file 1 [file Image_1.tif]
